# Maternal Burnout Syndrome: Contextual and Psychological Associated Factors

**DOI:** 10.3389/fpsyg.2018.00885

**Published:** 2018-06-05

**Authors:** Astrid Lebert-Charron, Géraldine Dorard, Emilie Boujut, Jaqueline Wendland

**Affiliations:** ^1^Laboratory of Psychopathology and Health Processes, Paris Descartes University, Paris, France; ^2^High School of Teaching and Education, University of Cergy-Pontoise, Paris Seine University, Cergy, France

**Keywords:** maternal burnout, associated factors, depression, anxiety, parenting stress, coping, perceived stress

## Abstract

**Background:** Becoming a parent is one of the most significant experiences in a woman’s life. Including substantial and long-lasting mental, social, and physical charge, the parenting experience may also be a potentially stressful and overwhelming task. Since the eighties, the notion of parental burnout syndrome has gained increasing attention, but its contextual and psychological factors need to be better identified.

**Aims:** To investigate a large array of contextual and psychological factors associated with maternal burnout syndrome in a French community-based population in order to contribute to better operationalize the notion of parental burnout and to explore its determinants.

**Method:** A total of 304 French-speaking mothers (mean age = 34.8 years, *SD* = 6.72) completed a set of questionnaires including a sociodemographic form (in order to gather general information about the mothers, their spouses, and children living at home). The Perceived Stress Scale, the Maslach Burnout Inventory adapted to parents (MBI-parental), the Hospital Anxiety and Depression Scale, the Parental Stress Index-Short Form and the Ways of Coping Checklist were used in this study.

**Results:** Multivariate linear regression analyses revealed that scores on the MBI-parental version were strongly and positively associated with depressive and anxiety symptoms, as well as with perceived stress related to parenthood and parenting stress levels. Moreover, using the task-oriented coping style in parenthood was strongly and positively associated with personal accomplishment. Conversely, some sociodemographic characteristics were found to be negatively associated with maternal burnout: being employed, working full time and being a mother living without a coparent.

**Conclusion:** The construct of maternal burnout syndrome seems to be linked to a conjunction of psychological and contextual factors associated with maternal exhaustion. The implication of the results for prevention and intervention strategies are discussed.

## Introduction

The notion of burnout was first defined in the professional realm as “the mental and physical exhaustion of working permanently in touch with others” (Maslach, 1976). It consists of an individual’s response when facing emotional stress and is characterized by a sequence of physical and psychological states (Perlman and Hartman, 1982). Two major conceptions predominate the literature. The first considers burnout as a unidimensional concept (Pines et al., 1981; Shirom and Melamed, 2006). The second considers the burnout syndrome as a multidimensional concept encompassing three dimensions: emotional exhaustion (a feeling of being emotionally overextended and depleted of emotional resources), cynicism or depersonalization (negative, indifferent or overly detached attitudes toward others) and loss of past and present accomplishments (declined feelings of competence and successful achievement) (Maslach, 1982).

The notion of maternal burnout stems from the concept of professional burnout and was first introduced by Freudenberger (1974) and Maslach and Jackson (1981). The authors found some similarities between professional exhaustion and the difficulties experienced by adults in their parental roles. They compared the workload of employees who worked permanently in contact with others to the amount of work associated to the parental task of child care (Maslach and Jackson, 1981). More recently, the notion of parental burnout has received growing attention and numerous narratives of mothers experiencing burnout have appeared in the mass media (Allenou, 2012; Eustache, 2016).

Parenthood is nowadays one of the most challenging tasks that adults face in their lives, often without any support, or preparation (Poole, 2003). Being a parent involves a significant mental and physical workload associated with different sources of stress and burden (Verjus and Boisson, 2005). The concept of maternal burnout has progressively emerged since the early eighties. A recent Belgian study estimated that approximately 8% of parents experience parental burnout (Roskam et al., 2017). However, this concept remains poorly identified and investigated. Only a few studies have explored its features on the basis of the similarities between professional burnout and the difficulties experienced by mothers (Freudenberger, 1974; Maslach, 1982; Pelsma et al., 1989; Le Vigouroux et al., 2017; Roskam et al., 2017; Mikolajczak et al., 2018). Furthermore, it remains unclear whether maternal burnout constitutes a new and unique clinical entity, distinct from maternal depression or anxiety.

Several factors associated with the parenting experience may contribute to the development of parental burnout, but they remain poorly explored. According to Abidin (1982), the stress experienced by a person in her/his parental role, designed as “parental stress,” is characterized by a limitation of the parent’s personal activities deriving from her/his continuous adaptation to her/his children’s demands and needs. Mothers, who are usually the primary caregivers (INSEE, 2012), are thought to be at greater risk in experiencing high levels of stress for many reasons. This huge and continuous responsibility might explain why women show poorer mental health when compared to men (Veroff et al., 1981). Similarly, Baruch et al. (1987) argued that women’s role in the family, which combine high levels of psychological demands with low levels of control, may generate high levels of stress. Parental stress levels are also higher since the mother is in charge of young children (Matthey, 2011). Having several children at home also increases the mothers’ feelings of stress (Lundberg et al., 1994). Moreover, the monotony of her endless daily tasks also leads to stress (Fisher, 1991). Finally, when mothers combine professional work and child care, they are at increased risk of experiencing not only high levels of stress, but also of anxiety or symptoms of depression (Naerde et al., 2000). Finally, Lacharité et al. (1992) postulate that raising and taking care of a child inevitably engenders a state of stress in individuals. This chronic state could contribute to the development of burnout syndrome (Zapf et al., 2001; Roskam et al., 2017).

In Europe, studies on parental burnout emerged in the 2000s, when exhaustion and worries of parents caring for children with acute and chronic health conditions were described. Norberg (2007) reported that Swedish mothers of children surviving brain tumors experienced symptoms of maternal burnout more frequently than mothers of children with no history of acute or chronic disease. In the same way, parents of children with chronic disease in Sweden (Lindström et al., 2010) and parents of children with cerebral palsy in Turkey (Basaran et al., 2013) felt significantly more parental burnout than parents of healthy children. Moreover, 34% of Swedish parents of children who underwent stem cell transplantation reported symptoms of burnout (Riva et al., 2014). Increased prevalence of burnout was also reported by mothers of children with Type 1 diabetes. The need for control was found to be associated with a sense of being constantly judged and feeling unsuccessful in mothers experiencing maternal burnout in Sweden (Lindström et al., 2017).

In a Belgian study, Mikolajczak et al. (2018) proposed a risk factor model for parental burnout. On the one hand, this model includes neuroticism, attachment avoidance, role restriction in parenthood, exposure to conflict in the couple, and family disorganization as risk factors for parental burnout. On the other hand, emotional intelligence, self-efficacy beliefs, positive parenting, coparental agreement, and marital satisfaction were found to be protective factors against parental burnout (Mikolajczak et al., 2018). Moreover, a French study on personality traits showed that parents having high levels of neuroticism and low levels of consciousness and agreeability were more likely to develop parental burnout symptoms (Le Vigouroux et al., 2017). However, other contextual and psychological factors inherent to parenting still need to be explored, such as the idealization of the parental role, the need to be perfect, the impact of the presence/absence of the child’s father, and the coping strategies employed in the specific context of parenting and parental burnout (Lebert et al., unpublished). Moreover, despite the abundant literature on the associations between professional burnout and depression (Schonfeld and Bianchi, 2016), to date, only one published study reported positive and significant correlations between depression and maternal emotional exhaustion scores (Roskam et al., 2017). Surprisingly, whereas depression and anxiety are strongly associated, to our knowledge, no study has explored anxiety symptoms in relation to maternal burnout.

In the 1980s, Lazarus and Folkman (1984) described the transactional model, which is one of the most robust models to understand the interplay between stress, coping, and health. This model describes stress as a process of interactions between the individual and his environment, named transactions. First, the individual assesses the situation and its characteristics as stressful or not. This model considers stress only within its subjective nature, namely perceived stress. The individual then assesses his own personal and social resources. To respond to his evaluation of the situation, the individual subsequently develops coping strategies. These coping strategies are applied to reduce the individual’s perceived stress of the situation. High levels of perceived stress require the individual to use adjustment strategies known as coping strategies (Folkman and Lazarus, 1988). Two major types of coping strategies are defined: problem-focused coping and emotion-focused coping. The first contributes by decreasing the emotional tensions experienced by the individual by focusing on the source of stress, whereas the second tries to act on the emotional signs induced by the situation (Bruchon-Schweitzer, 2001). Emotion-focused coping strategies may lead to psychological symptoms, such as depressed mood and emotional distress (McWilliams et al., 2003).

In a recent study, mothers’ parenting stress in the context of child disability was found to be positively correlated with emotional-oriented coping and negatively correlated with task-oriented coping (Najmi et al., 2017). Furthermore, using social support coping strategies contributes to reduce parental stress in parents of children with disabilities, while not using social support strategies may result in negative behavior of parents (Jones and Passey, 2005). However, parents of children with autism or Down syndrome more commonly use emotional-oriented coping, while parents of typical children mostly use task-oriented coping (Dabrowska and Pisula, 2010).

Primiparous mothers are at greater risk of perceiving high levels of stress during the transition to parenthood (Campbell-Grossman et al., 2009). High levels of perceived stress in mothers were found to be often related to depressive symptoms (Mora et al., 2009; Manuel et al., 2012; Campbell-Grossman et al., 2016). Israel et al. (2002) found that receiving emotional support (defined as the provision of affection, listening, liking, and love; House, 1981) contributes to reduce perceived stress in mothers of young children. Indeed, in the family context, the perception of having adequate social support leads to reduced stress and tends to improve positive feelings linked to parenthood (Lyons et al., 2005). For example, the well-being of mothers caring for a child with autism is predicted by the quality of her perceived social support (Smith et al., 2012). In addition, support from school, childcare staff, and friends may help mothers of chronically ill children to feel better, dispelling stress, and relinquishing control (Lindström et al., 2017). Although single mothers have, in general, to face more stressful living conditions, they do not necessarily perceive higher levels of stress than non-single mothers, as they may experience being able to manage and to raise their children on their own (Son and Bauer, 2010).

Thus, given the recent attention paid to this concept and the paucity of studies focusing on factors associated with maternal burnout, the present study aims at extending the understanding of this phenomenon. While there is a growing body of studies specifically centered on maternal burnout among mothers of children with illness, only a few studies have investigated maternal burnout in the general population. Moreover, the transactional model (Lazarus and Folkman, 1984) has demonstrated the relevance of perceived stress and coping to predict professional burnout, these notions have never been studied in the context of maternal burnout. In addition, the links between depression and burnout have not yet been clearly explored in the context of parenting and the links between anxiety and parental burnout have never been studied. However, the relationships between anxiety, depression, and burnout are well established in the professional context. Moreover, contextual dimensions such as “employment,” “working time,” and “parent status” are relevant in professional burnout and parenting studies but have not yet been explored in the context of maternal burnout.

Therefore, the aims of this study are to: (1) explore some contextual factors associated with maternal burnout (employment, working time, and parent status), and (2) explore some of the psychological factors associated with maternal burnout (anxiety, depression, perceived stress, parenting stress, and coping).

## Materials and Methods

### Participants and Procedure

Participants were 304 French-speaking mothers recruited from the general population, aged more than 18 years old, and having a child living regularly at home.

An announcement presenting the research on “the experience of parenting” was posted on several social networks, after receiving an authorization from the website administrators. The announcement was also sent by email to the private social networks of the investigators and an additional message asked the participants to forward the request to their friends to create a “snowball sampling.” Persons interested in participating were directed to an URL on Google form. An information form describing the objectives and the procedures of the study and providing contact details of the study investigators was first provided to the participants. All participants had to give their consent before they had access to the questionnaires. At the end of the questionnaires, contact data of the investigators were given once again. Participants were also invited to seek psychological support from their local public mental health services if they felt any discomfort in answering the questionnaires. The study protocol was approved by the local ethical committee of Paris Descartes University (IRB: 20162900001072). The design of the study and the data collection took place between February 2016 and April 2016.

The questionnaires were completed online by mothers in approximately 30 min and a forced choice procedure prevented missing data. Inclusion criteria were: being a French-speaking mother aged more than 18 years old and having at least one child living at home regularly. No participant was excluded.

### Instruments

Participants completed a set of self-report questionnaires including:

#### An *Ad hoc* Sociodemographic Questionnaire

Participants were asked about their parent status (mothers living without a coparent, mothers living with a coparent, and mothers having a coparent but living apart), their age, the duration of their relationship, the number of children living with them, the children’s characteristics (age, health status, as declared by mothers), their work status (employed, housewife, and unemployed), and the working time of employed mothers.

#### The Perceived Stress Scale (PSS)

The French version of the Perceived Stress Scale (PSS) (Cohen et al., 1983; Quintard, 1994) was used. Based on the transactional model of stress, the PSS is intended to identify the psycho-cognitive mechanisms of stress. The PSS comprises 14 items answered on a 5-point Likert scale (from 0: “never” to 5: “always”) that evaluates the frequency of everyday life situations perceived as threatening over the previous month (e.g., “how often have you felt nervous and stressed?”). In the current study, Cronbach’s alpha is satisfactory (0.85).

#### The Maslach Burnout Inventory Adapted to Parenthood (MBI-P)

Maternal burnout was measured with the Maslach Burnout Inventory (MBI – Maslach and Jackson, 1981) adapted to parenthood (MBI-P) by the researchers, to evaluate the intensity of maternal burnout. The instructions of the questionnaire were reformed into «my child» instead of «my recipients», and «being a mother» instead of «my jo»b or «my work». This scale consists of 22 items answered on a seven-point Likert scale (from 0: “never” to 6: “every day”). As the original version, the MBI-P comprises three subscales: emotional exhaustion (MBI-P EE; e.g., “I feel emotionally drained from my children”); depersonalization (MBI-P D; e.g., “I feel my children blame me for some of their problems”); and decrease of personal accomplishment (MBI-P PA; e.g., “I can effectively solve the problems that arise to my children”). In the present study, Cronbach’s alphas are satisfactory, except for the depersonalization sub-dimension (MBI-P EE = 0.92, MBI-P D = 0.51, and MBI-P PA = 0.78).

#### The Hospital Anxiety and Depression Scale (HADS)

The French version of the Hospital Anxiety and Depression Scale (HADS) measuring depression and anxiety symptoms (Zigmond and Snaith, 1983; Lépine et al., 1985) was used. This scale comprises 14 items divided into two subscales that assesses the presence and the intensity of anxious symptoms (HADS-A; e.g., “I feel tense or ‘wound up”’) and depressive symptoms (HADS-D; e.g., “I feel as if I am slowed down”). Participants answer on a 4-point Likert scale ranging, for each item, from “3: most of the time” to “0: not at all”, or from “3: nearly all the time to 0: not at all.” In the present study, Cronbach’s alphas are satisfactory for the two dimensions (0.78 and 0.77, respectively).

#### The Parenting Stress Index – Short-Form (PSI-SF)

The French version of Parenting Stress Index-Short-Form (PSI-SF; Abidin, 1983; Bigras et al., 1996), a shorter version of the Parenting Stress Index created by Abidin (1983), was used in order to assess parenting stress. The PSI-SF consists of 36 items divided into three subscales: Parental Distress (e.g., “I often have the feeling that I cannot handle things very well”); Parent – Child Dysfunctional Interaction (e.g.,“ My child rarely does things for me that make me feel good”), and Difficult Child (e.g., “My child seems to cry or fuss more often than most children”). A modified instruction was used to evaluate the parental stress of mothers who have several children. They had to answer by considering all their children as a whole. Scores can be calculated for each subscale, while the total score reflects the intensity of the stress generated by the parent–child relationship. Lower scores indicate less stress. Participants answer on a 5-point Likert scale (from 5: “strongly agree” to 1: “strongly disagree”). Only the total score was used in the present study. Cronbach’s alpha for the global score is excellent (0.92).

#### Ways of Coping Checklist (WCC-R)

The French version of the Ways of Coping Checklist-Revised (WCC-R; Vitaliano et al., 1985; Cousson-Gélie et al., 1996) consists of 27 descriptions of coping strategies answered on a 4-point Likert scale (from 1: “no” to 4: “yes”). A situational version adapted to parenting was used: participants were asked to think about a stressful situation related to their maternal experience. The WCC comprises three factors: emotion-focused coping (WCC-E; e.g., “Hoped a miracle would happen”), problem-focused coping (WCC-P; e.g., “Stood my ground and fought for what I wanted.”), and social support seeking (WCC-S; e.g., “Talked to someone to find out more about the situation”). Cronbach’s alphas are satisfactory (i.e., 0.74, 0.80, and 0.78, respectively).

### Data Analysis

Descriptive statistics for quantitative measures (mean, variance, standard deviation) and for qualitative measures (percentage) were first calculated. Analyses of variances (ANOVAs) with Tukey *post hoc* were then performed to estimate group effects of three selected variables (mother’s employment, working time, and parent status) on the three maternal burnout dimensions (emotional exhaustion, depersonalization, and decrease of personal accomplishment). Employment data was coded into three groups: mothers who declared being at home (housewife), mothers who were employed, and mothers who were unemployed. Regarding working time, employed mothers were separated into four groups: mothers working full time, mothers working more than part-time, mothers working part-time, and mothers working less than part-time. The parent status sample was divided into three groups: mothers living without a coparent, mothers living with a coparent, and mothers having a coparent but living apart.

Additionally, multivariate linear regressions were carried out to test the predictive effect of depression and anxiety symptoms, problem-focused coping strategies, emotion-focused coping strategies, social support seeking strategies, parenting stress, and perceived general stress scores on the three dimensions of maternal burnout (emotional exhaustion, depersonalization, and decrease of personal accomplishment). Three distinct models were calculated for each MPI-P dimension score as a dependent variable.

All analyses were performed with SPSS-21 software and hypotheses were tested with a two-sided significance level of 0.05.

## Results

### Sample Characteristics

Data were collected from a sample of 304 mothers aged from 18 to 60 years old (*M* = 34.8, *SD* = 6.7). Sociodemographic and psychometric characteristics of the participants are described in **Table 1**. Regarding parent status, 89.1% of mothers lived with a coparent (*n* = 271), 5.9% had a coparent but lived apart (*n* = 18), and only 4.9% had no coparent (*n* = 15). The mean duration of cohabitation was 11.13 years (*SD* = 5.9). Children’s mean age was 6.9 years old (*SD* = 6.0, [0; 37]), and mothers had on average 2.15 children (*SD* = 1.3). Regarding work status, most mothers (63.5%) were employed (*n* = 193), 11.2% were unemployed (*n* = 34), and 25.3% were housewives (*n* = 77). More than half of employed mothers had a full-time job (52.5%; *n* = 96).

**Table 1:** Descriptive characteristics of the sample (*N* = 304).

	Mothers *N* (%) Mean (*SD*)
**Mothers**	
Age	34.84 (6.72)
**Parent Status**	
Mothers living without a coparent	15 (4.93%)
Mothers living with a coparent	271 (89.14%)
Mothers with a coparent but living separated	18 (5.92%)
**Mothers**	
Duration of common Life	11.13 (5.86)
**Children**	
Number of children	2.15 (1.3)
Age of children	6.9 (6.02)
Sick children	58 (8.71%)
**Employment**	
Employed	193 (63.49%)
Unemployed	34 (11.18%)
Housewife	77 (25.33%)
**Working time of employed mothers (*N* = 193)^∗^**	
Full time	96 (52.46%)
More than part-time	55 (30.05%)
Part-time	22 (12.02%)
Less than part-time	10 (5.46%)
**Burnout (MBI-P)**	
Emotional exhaustion (MBI-P EE)	23.18 (13.4)
Depersonalization (MBI-P D)	5.2 (4.86)
Personal accomplishment (MBI-P PA)	37.16 (7.43)
**Coping (WCC)**	
Problem-focused (WCC-P)	29.93 (4.31)
Emotion-focused (WCC-E)	23.96 (4.73)
Social support seeking (WCC-S)	21.82 (4.44)
Depression (HADS-D)	7.17 (4.14)
Anxiety (HADS-A)	9.86 (4.2)
Perceived stress (PSS)	30.6 (6.25)
Parental stress (PSI)	90.32 (20.46)

*SD, Standard Deviation; HAD-A, Hospital Anxiety and Depression Scale, Anxiety dimension; HAD-D, Hospital Anxiety and Depression Scale, Depression dimension; MBI-P EE, Maslach Burnout Inventory adapted to parenthood, Emotional Exhaustion dimension; MBI-P D, Maslach Burnout Inventory adapted to parenthood, Depersonalization dimension; MBI-P PA, Maslach Burnout Inventory adapted to parenthood, Personal Accomplishment dimension; PSI, Parental Stress Index; PSS, Perceived Stress Scale; WCC-E, Ways of Coping Checklist, Emotion-focused coping dimension; WCC-P, Ways of Coping Checklist, problem-focused coping dimension; WCC-S, Ways of Coping Checklist, seeking social support dimension. ^∗^10 missing data.*

### Comparison of Maternal Burnout Scores According to Contextual Variables

The effect of three independently selected contextual variables (i.e., mother’s employment, working time, and parent status) on burnout dimensions was tested through ANOVAs (**Table 2**). Some overall effects were found for emotional exhaustion score (MBI-P EE), but not for depersonalization (MBI-P D), nor for decrease of personal accomplishment (MBI-P PA) scores. *Post hoc* Tukey analyses revealed that emotional exhaustion scores of employed mothers were significantly lower than those of housewives [mean difference (MD) = 5.85, *p* < 0.001]. Furthermore, emotional exhaustion scores of employed mothers working full-time were significantly lower than those of employed mothers working more than part-time (MD = 6.06, *p* < 0.001), and part-time (MD = 8.09, *p* < 0.001). Finally, emotional exhaustion scores of mothers living without a coparent were significantly lower than those of cohabiting mothers (MD = -8.99, *p* = 0.03).

**Table 2:** Comparison of group means (ANOVAs) for contextual variables and maternal burnout (MBI-P).

	Employment						Working time							Parent status					
	Employed 1 *N* = 193	Housewife 2 *N* = 77	Unemployed 3 *N* = 34				Full time 1 *N* = 96	More than part-time 2 *N* = 55	Part-time 3 *N* = 22	Less than part-time 4 *N* = 10				Living without a coparent 1 *N* = 15	Living with a coparent 2 *N* = 271	With a coparent but living separated 3 *N* = 18			
			
	Mean (*SD*)	Mean (*SD*)	Mean (*SD*)	*F*	*df*	*p*	Mean (*SD*)	Mean (*SD*)	Mean (*SD*)	Mean (*SD*)	*F*	*df*	*p*	Mean (*SD*)	Mean (*SD*)	Mean (*SD*)	*F*	*df*	*p*
Emotional Exhaustion (MBI-P EE)	21.21 (12.43)	27.06 (14.54)	25.53 (14.13)	6.03	2;301	<0.001^a^	18.05 (11.13)	24.56 (13.24)	26.14 (11.85)	20.20 (14.12)	4.66	3;199	0.01^b^	14.67 (9.43)	23.66 (13.57)	23.06 (11.74)	3.25	3;300	0.04^c^
Depersonalization (MBI-P D)	4.76(4.48)	6.19(5.64)	5.47(4.84)	2.47	2;301	0.09	4.65 (4.71)	4.91 (4.30)	5.09 (4.60)	6.1(4.53)	0.16	3;199	0.92	4.13 (2.61)	5.21 (4.95)	6.06 (5.02)	0.64	3;300	0.53
Personal Accomplishment (MBI-P PA)	36.87 (7.39)	37.65 (7.79)	37.71 (6.93)	0.41	2;301	0.66	37.39 (37.39)	36.60 (7.00)	33.45 (9.16)	38.30 (5.81)	2.31	3;199	0.08	38.27 (7.43)	37.24 (7.20)	34.94 (10.52)	0.98	3;300	0.38

*^*a*^Post hoc: 1<2, ^*b*^Post hoc: 1<2; 1<3, ^*c*^Post hoc: 1<2. MBI-P EE, Maslach Burnout Inventory adapted to parenthood, Emotional Exhaustion dimension; MBI-P D, Maslach Burnout Inventory adapted to parenthood, Depersonalization dimension; MBI-P PA, Maslach Burnout Inventory adapted to parenthood, Personal Accomplishment dimension.*

### Multivariate Linear Regression Analysis With Psychological Variables Predicting Maternal Burnout Scores

Three models of multivariate linear regression analysis were performed with the three maternal burnout dimensions (emotional exhaustion, depersonalization, and decrease of personal accomplishment) as dependeport seeking, depression and anxiety symptoms, perceived stress and parenting stress as independent variables (**Table 3**).

**Table 3:** Multivariate linear regressions with psychological variables predicting maternal burnout scores (MBI-P).

Dependent variables	Independent variables	β	*p*	Adjusted *R*^2^	*F*	*p*
Emotional Exhaustion (MBI-P EE)	Problem-focused (WCC-P)	0.03	0.5	0.63	65.17	<0.001
	Emotion-focused (WCC-E)	0.01	0.86			
	Social support seeking (WCC-S)	0.02	0.03			
	Depression (HADS-D)	0.25	<0.001			
	Anxiety (HADS-A)	0.18	<0.001			
	Perceived stress (PSS)	0.25	<0.001			
	Parenting stress (PSI)	0.29	<0.001			
Depersonalization (MBI-P D)	Problem-focused (WCC-P)	0.12	0.06	0.24	13.04	<0.001
	Emotion-focused (WCC-E)	0.12	0.07			
	Social support seeking (WCC-S)	-0.09	0.18			
	Depression (HADS-D)	0.21	0.01			
	Anxiety (HADS-A)	0.25	0.73			
	Perceived stress (PSS)	-0.04	0.62			
	Parenting stress (PSI)	0.36	<0.001			
Personal accomplishment (MBI-P PA)	Problem-focused (WCC-P)	0.32	<0.001			
	Emotion-focused (WCC-E)	-0.05	0.42	0.37	23.07	<0.001
	Social support seeking (WCC-S)	-0.02	0.69			
	Depression (HADS-D)	-0.05	0.49			
	Anxiety (HADS-A)	-0.01	0.99			
	Perceived stress (PSS)	-0.13	0.05			
	Parenting stress (PSI)	-0.24	<0.001			

*HAD-A, Hospital Anxiety and Depression Scale, Anxiety dimension; HAD-D, Hospital Anxiety and Depression Scale, Depression dimension; MBI-P EE, Maslach Burnout Inventory adapted to parenthood, Emotional Exhaustion dimension; MBI-P D, Maslach Burnout Inventory adapted to parenthood, Depersonalization dimension; MBI-P PA, Maslach Burnout Inventory adapted to parenthood, Personal Accomplishment dimension; PSI, Parenting Stress Index; PSS, Perceived Stress Scale; SSQ-6, Social Support Questionnaire 6, Satisfaction dimension; WCC-E, Ways of Coping Checklist, Emotion-focused coping dimension; WCC-P, Ways of Coping Checklist, problem-focused coping dimension; WCC-S, Ways of Coping Checklist, seeking social support dimension.*

The first model with emotional exhaustion score as dependent variable explained 63% of the MBI-P EE score variance. More precisely, anxiety (β = 0.18, *p* < 0.001), depression (β = 0.25, *p* < 0.001), parenting stress (β = 0.29, *p* < 0.001), and perceived stress (β = 0.25, *p* < 0.001) scores positively and significantly predicted the emotional exhaustion score.

The second model, with depersonalization score as dependent variable explained 24% of the MBI-P D score. Depression (β = 0.21, *p* = 0.01) and parenting stress (β = 0.36, *p* < 0.001) scores positively and significantly predicted the depersonalization score.

The third model, with personal accomplishment score as dependent variable explained 37% of the variance of the MBI-P PA score. Parenting stress score (β = -0.24, *p* < 0.001) was negatively associated with personal accomplishment score, whereas problem-focused coping (β = 0.32, *p* < 0.001) had a significant and positive relationship with personal accomplishment score.

## Discussion

The aims of this study were to explore some contextual and psychological factors associated with maternal burnout. Overall, our results suggest that being employed, working full-time, living without a coparent, and using problem-focused coping are negatively associated with maternal burnout. Conversely, in accordance with previous studies, depressive and anxiety symptoms, as well as high levels of parenting stress and perceived stress are positively associated with maternal burnout.

First, regarding work status, this study highlights that being employed is negatively linked with maternal burnout and in particular with maternal emotional exhaustion. In fact, employed mothers feel less emotionally exhausted than housewives, but this link is not significant with other burnout dimensions, namely depersonalization and decrease of personal accomplishment assessed in a parental context. This result is consistent with the recent study of Mikolajczak et al. (2018) who found that unemployed parents had a higher risk of parental burnout. Along with the Mikolajczak et al. (2018) study, this is the first study that shows a significant link between maternal employment status and burnout. This result is in line with the Guéritault (2004) hypothesis which proposed that housewives would be more likely to develop maternal burnout than employed mothers. Their vulnerability could be explained by a lack of social recognition of their roles (Skinner, 1985), their low levels of control (Karasek, 1989), and the work overload associated with an absence of fulfillment (Bensahel, 1978).

Moreover, both work status and family roles are in continuous interaction (Vidanovic et al., 2006), as job satisfaction is known to have a positive influence on life satisfaction (van Praag et al., 2003). Employed women tend to also be more satisfied in their marital relationship than unemployed women, probably because they feel more equity with their spouse (Bahmani et al., 2013). Interestingly, our results also reveal that employed mothers working full time feel less emotionally exhausted than mothers working part-time, or more than part-time. Thus, beyond the fact of being employed, the amount of time spent in work also has a significant impact on maternal emotional exhaustion. This finding is consistent with Mikolajczak et al. (2018) study which also found that mothers working part-time were at greater risk of parental burnout compared to those working full time. This could be explained by the amount of time mothers who work less than full time spend with their children (Zick and Bryant, 1996). However, these hypotheses need to be empirically tested in future studies with a precise assessment of the time spent with children and taking into account if the employment status (part- or full-time job or being housewife) is a voluntary choice of the mothers or not.

Parent status was also found to have significant links with maternal burnout. Our results show that mothers living without a coparent feel less emotionally exhausted than cohabiting mothers. At first sight, this result is surprising given that single mothers are expected to be at greater risk of maternal burnout. Mikolajczak et al. (2018) and Lebert et al. (unpublished) suggested that being single would increase parents’ vulnerability to parental burnout, but this was not confirmed. Many studies have shown that single mothers or mothers living with a coparent are more likely to feel depressed (Peden et al., 2004), to perceive higher levels of stress (Son and Bauer, 2010), to be exposed to stressful life conditions such as financial difficulties (Kotwal and Prabhakar, 2009), to be at higher risk of poverty (Mistra et al., 2012), and to feel isolated or unsatisfied in their life (Lipman et al., 2010). Single mothers usually have to deal with both domestic and professional work without any support from a spouse. However, because everyone knows that being a single mother is a hardship, these mothers may receive more support from their close relatives than women living in couples. Stack (1974) observed that single mothers may have a strong social network helping them. We can also hypothesize that single mothers may be less emotionally exhausted than cohabiting mothers in that they may be “protected” from relationship dissatisfaction and challenges of living as a couple. In fact, when married working women perceive inequality vis-à-vis the household labor, they are more vulnerable to developing depression (An et al., 2016). Forthcoming studies could investigate the links between satisfaction with couple relationship and maternal burnout. However, these results must be considered with caution given that single mothers represent only 4.9% of our sample, but are 8.9% of the French general population (French National Institute of Statistics, and Economical Studies [NISES], 2013). The links between parent status, couple’s relationship, and maternal burnout need to be further explored with a wider population, including a more representative distribution of family situations.

The issue of independence between depression and burnout remains controversial. In the parental context, only one study (Roskam et al., 2017) has explored the links between these two dimensions and has shown significant and positive correlations between depression and emotional exhaustion, and between depression and decrease of personal accomplishment. In the same way, the present study confirms these associations in the context of maternal burnout. Our results show that high levels of depression in mothers are associated with high levels of emotional exhaustion and depersonalization.

This is the first study to explore the relationship between anxiety and burnout in the parental context. Although anxiety and depression are closely related, we deemed to be relevant to explore depressive and anxiety symptoms separately. Our results showed that high levels of anxiety are associated with high levels of emotional exhaustion, but not with the other dimensions of maternal burnout. These results are in line with a recent study on professional burnout that showed that both depressive and anxiety symptoms are common predictors of emotional exhaustion and depersonalization (Lee et al., 2018). However, our results did not confirm the links previously observed, in the professional realm, between anxiety symptoms and emotional exhaustion and depersonalization dimensions (O’Mahony, 2011; Stathopoulou et al., 2011), or the three burnout components (Astres Fernandes et al., 2012; Jiang et al., 2017). Thus, maternal anxiety seems to differ from maternal burnout given its association with emotional exhaustion but not with the two other dimensions. This may be explained by the fact that being anxious may deplete personal resources and lead to exhaustion, but not to depersonalization or decrease of personal accomplishment.

Studies’ results are not convergent on this point. While Roskam et al. (2017) found that depression was correlated with two dimensions of parental burnout (emotional exhaustion and decrease of personal accomplishment), another recent study on professional burnout showed a reverse direction for the relationship between anxiety, depression, and burnout: High emotional exhaustion and high depersonalization were found to be associated with an increased risk of depressive and anxiety symptoms (Lebares et al., 2018). This latter idea is in line with other studies that support the idea that professional burnout plays an important role in the occurrence of anxiety and depression (Ahola and Hakanen, 2007; Ahola et al., 2008; Papathanasiou et al., 2017). According to Soares et al. (2007), the distinction between burnout and depression is not clear because of their correlated scores. In our study, depression was found to be significantly associated only with two dimensions of maternal burnout. These results tend to support the assumption that depressive symptoms and maternal burnout are two distinct concepts (Mikolajczak et al., 2018; Lebert et al., unpublished). Moreover, the links between depression and depersonalization could be explained by the fact that some severe depressive symptoms may be associated with depersonalization, such as melancholic symptoms. Depressive mothers tend to be insensitive and detached when caring for their children (Smith-Nielsen et al., 2016). Mother’s insensitivity and depersonalization could be considered as two close concepts.

In the present study, perceived stress was found to be associated with high emotional exhaustion and low personal accomplishment in mothers. According to the transactional model, when a person perceives stress, he/she uses coping strategies to reduce the stress experience. But if stress is not reduced, this could lead to poor psychological adjustment and to burnout (Boujut et al., 2016). Thus, our findings seem to be consistent with the transactional model of stress (Lazarus and Folkman, 1984). Whereas we refer to the theoretical transactional model of stress of Lazarus and Folkman (1984), and we evaluate two of its key dimensions (i.e., perceived parental stress and coping strategies), we did not test the model. This could be the objective of future studies on maternal burnout in order to achieve a better understanding of the concept and its associations with stress. Recently, Lee et al. (2018) found that depression and anxiety symptoms are associated with both perceived professional stress and professional burnout. However, while Koleck et al. (2000) showed that perceived stress, but not the workload, lead to professional burnout, Evers et al. (2002) argued that workload is a major cause of professional burnout. In turn, Laugaa (2004) found that professional burnout could be a consequence of perceived stress. Thus, it is plausible that relationships between these notions are somewhat distinct in the context of parental burnout.

In line with the above results, high levels of parenting stress were associated with high levels of emotional exhaustion and depersonalization and low levels of personal accomplishment. These results are consistent with those from Roskam et al. (2017) who demonstrated that parenting stress was positively correlated with the three dimensions of their Parental Burnout Inventory (PBI): emotional exhaustion, emotional distancing, and decrease of personal accomplishment. It is widely accepted that professional burnout emerges, in part, when perceived stress is persistent. The association between parenting stress and maternal burnout seems to be similar to the association between professional stress and professional burnout. Many studies have demonstrated that professional stress is associated with the three dimensions of professional burnout (Zapf et al., 2001; Garrosa et al., 2008; Guo et al., 2016). In the present study, stress related to parenthood was assessed with a specific parenting stress tool. So, it seems cogent that parenting stress was found to be significantly linked with all three dimensions of maternal burnout. However, in line with Roskam et al. (2017) and the review of literature from Lebert et al. (unpublished) our results support the notion that maternal burnout is not only parenting stress.

To our knowledge, this is the first study which has investigated the effect of coping strategies related to burnout syndrome in the parenting context. The transactional model of stress seems to be appropriately adapted to explain maternal burnout in our study, given the significant associations between perceived stress and coping strategies, and maternal burnout. Our results show that when mothers used predominantly problem-focused coping strategies in a parental context they have higher levels of personal accomplishment. Problem-focused coping is one of the main adjustment strategies to help face stress (Laugaa and Bruchon-Schweitzer, 2005), because this strategy is intended to reduce or to remove the stressful situation and to diminish perceived stress. In the professional domain, several studies have shown a positive association between the use of problem-focused coping and personal accomplishment (Shin et al., 2014; Foley and Murphy, 2015; Lee et al., 2016). According to van der Colff and Rothmann (2014), when individuals use problem-focused coping strategies they can manage the stressful situation by themselves, which in turn increases the feeling of personal accomplishment (Lee et al., 2016). The use of problem-focused coping strategies by nurses was found to be associated with a decrease in burnout symptoms (Li et al., 2014; Shin et al., 2014). As mentioned above, our study is the first to investigate the effect of specific coping in a parental context on maternal burnout. The instructions in the Ways of Coping Checklist were adapted to parenthood in order to measure coping in this specific context. In previous studies on parent–child relationships, problem-focused coping strategies were found to lead to lower levels of parenting stress (Mu et al., 2005; Rodenburg et al., 2007). Moreover, it has also been shown that mothers who use problem-focused coping strategies tend to adopt healthier behaviors with their children (Matsuo and Sato, 2017). Thus, our results suggest that problem-focused coping is more functional than emotion-focused coping for mothers when dealing with their children. With regard to burnout dimensions, no association was found with the emotional exhaustion scores which appear to be the core dimension of burnout in the maternal context (for professional context see: Pines et al., 1981). These results on coping adjustment deserve further replication.

It should be noted that almost all the independent variables (employment, working time of employed mothers, parent status, depressive and anxiety symptoms, parenting stress and perceived stress) considered in the current research were associated with emotional exhaustion. The internal consistency of this dimension is the highest of the three dimensions of MBI-P (α = 0.92). In contrast, depersonalization has only a moderate reliability in our sample (α = 0.51). The poor reliability of this dimension was also reported by Roskam et al. (2017) in their validation study of the PBI. These two recent results taken together lead to the question of the relevance of the depersonalization dimension when evaluating burnout in the parental context. Moreover, this moderate internal consistency is in line with previous studies using the original MBI version (Kantas and Vasilaki, 1997). Dion and Tessier (1994), in the French validation of the Maslach Burnout Inventory, also found a moderate reliability for the depersonalization dimension (α = 0.64). Thus, our results on depersonalization must be considered with caution. In turn, the high internal consistency of emotional exhaustion supports the notion of burnout as a unidimensional concept. Pines et al. (1981) and Shirom and Melamed (2006) argued that burnout is an emotional, mental, and physical state of exhaustion which does not include the dimensions of depersonalization and decrease of personal accomplishment. In fact, emotional exhaustion is usually considered as the central concept of burnout (Taris et al., 2005) and our results corroborate this assumption.

This study has several limitations. First, a non-validated questionnaire for maternal burnout was used. When we started this study, no validated tool for the assessment of maternal burnout was available. Secondly, the recruitment approach, based on an online survey, did not allow us to estimate the participation and refusal rates, nor to obtain a more representative sample of French mothers’ general population. In fact, we have very few mothers living without a coparent. Moreover, given the subject of the research presented in the information sheet, it is possible that mothers who participated were seeking some kind of support and an opportunity to share their feelings of dissatisfaction, exhaustion, or depression, thus resulting in some recruitment bias. In addition, it would be interesting in future studies to collect other contextual information on educational levels, social status and types of professions held by women as these variables could be related to their job satisfaction. Moreover, the ages of the mothers and the children, as well as the number of children, should be considered in future studies on their relationships with maternal burnout dimensions. Finally, a longitudinal approach would be welcome in order to verify the stability of contextual and psychological factors involved in maternal burnout.

## Conclusion

In conclusion, this study shows that a substantial number of mothers in the general population live under high levels of stress related to their daily parental workload. Interestingly, our results suggest that being employed, and notably working full-time, living without a coparent and using problem-focused coping strategies are associated with lower levels of maternal burnout. Conversely, depression, anxiety, parenting stress, and perceived stress are strongly and positively associated with maternal burnout. In addition, the transactional model of stress seems to be appropriately adapted to explain maternal burnout given the positive associations found in perceived stress and coping with maternal burnout.

Identifying maternal burnout contextual and psychological factors would enable a more precise profile of vulnerable mothers. It also opens the possibility to set up therapeutic strategies, such as psychoeducation groups for mothers suffering from maternal burnout. Prevention of maternal burnout should also be one of the next steps of future research.

## Author Contributions

AL-C designed the study, performed the recruitment of participants and the statistical analysis, and wrote the paper. GD designed the study, supervised the recruitment of participants and the statistical analysis, provided guidance and oversight throughout the project, and contributed to writing, proofreading, and editing the final paper. EB designed the study, provided guidance and oversight throughout the project, and contributed to writing, proofreading, and editing the final paper. JW designed the study, provided guidance and oversight throughout the project, and contributed to writing, proofreading, and editing the final paper.

## Conflict of Interest Statement

The authors declare that the research was conducted in the absence of any commercial or financial relationships that could be construed as a potential conflict of interest.
